# Nutrient addition and herbivore exclusion alter plant traits and biomass via distinct mechanisms: intraspecific variability vs species turnover

**DOI:** 10.1111/nph.70827

**Published:** 2025-12-17

**Authors:** Xuebin Yan, Risto Virtanen, Anu Eskelinen

**Affiliations:** ^1^ Ecology and Genetics Unit University of Oulu P.O. Box 3000 Oulu Finland; ^2^ College of Resources and Environmental Sciences Nanjing Agricultural University Nanjing 210095 China; ^3^ German Centre for Integrative Biodiversity Research (iDiv) Halle‐Jena‐Leipzig 04103 Leipzig Germany

**Keywords:** functional trait dimension, grazing, intraspecific trait, nutrient enrichment, trait identity

## Abstract

- Soil nutrients and vertebrate herbivory are key ecological factors with opposite and interactive effects on grassland plant traits and biomass. Partitioning trait changes into species turnover and intraspecific change provides a mechanistic linkage between trait shifts and biomass responses. However, their relative contributions in determining plant responses to nutrients and herbivory remain unclear.
- Based on a long‐term experiment in two grasslands differing in productivity, we examined how nutrient addition and herbivore exclusion influenced plant functional composition and biomass, and quantified contributions of inter‐ and intraspecific trait change.
- Nutrient addition shifted leaf economics traits to be faster‐growing and increased plant height, while herbivore exclusion boosted height and leaf area, both mainly through intraspecific changes. These effects were habitat‐dependent: leaf economics traits dominated in the low‐productivity grassland, while size‐related traits prevailed in the high‐productivity grassland. Nutrient addition and herbivore exclusion had weak effects on plant defense traits (tannins). Biomass responses to nutrient addition and herbivore exclusion were, to a greater extent, associated with intraspecific trait variation than species turnover.
- This study highlights how partitioning traits into different dimensions helps understand the distinct pathways through which nutrients and herbivores shape plant communities, how these vary across environments, and ultimately influence ecosystem functioning.

## Introduction

Soil nutrients and vertebrate herbivores are two important and counteracting ecological factors that control plant biomass in grassland ecosystems (McNaughton *et al*., 1989; Fay *et al*., 2015; Jia *et al*., 2018; Borer *et al*., 2020). Anthropogenic activities have caused elevated nutrient inputs and alleviated nutrient limitation in terrestrial ecosystems (Borer & Stevens, 2022), thus increasing plant biomass (Elser *et al*., 2007; Isbell *et al*., 2013). Conversely, food‐limited herbivores, especially mammalian herbivores, can substantially reduce plant biomass (Eskelinen *et al*., 2012; Jia *et al*., 2018; Borer *et al*., 2020; Zheng *et al*., 2022). Therefore, nutrients and herbivores are predicted to interact to influence plant biomass (Huisman *et al*., 1999; Bakker *et al*., 2006), yet there is no consensus about the magnitude of these interactions and which mechanisms underlie them (Borer *et al*., 2020).

Trait‐based approaches can reveal mechanisms underlying the effects of nutrient addition and herbivory on plant biomass (Díaz *et al*., 2016; Jia *et al*., 2018; Firn *et al*., 2019; Pichon *et al*., 2022). Generally, plant aboveground traits vary along two major axes (Díaz *et al*., 2016). The first axis is the leaf economics spectrum, which distinguishes fast‐growing plants with acquisitive resource‐use strategies from slow‐growing plants with conservative resource‐use strategies (Wright *et al*., 2004; Reich, 2014). The other axis is related to plant size, including plant height and leaf area, which characterizes the trade‐offs between investment in competition for light, photosynthesis, and reproduction (Díaz *et al*., 2016). Nutrient addition typically shifts plant community composition towards the dominance of fast‐growing species, indicated by increased specific leaf area (i.e. SLA), decreased leaf dry matter content (i.e. LDMC), and leaf carbon : nitrogen ratio (i.e. C : N) (Eskelinen *et al*., 2012; Tatarko & Knops, 2018; Firn *et al*., 2019). Also, nutrient addition‐induced light limitation favors the dominance of tall and large‐leaved plants (DeMalach *et al*., 2016; Xiao *et al*., 2021). However, changes in both leaf economics spectrum and size‐related traits can also make plants more vulnerable to herbivores, which may lead to increased biomass loss in grazed conditions (Díaz *et al*., 2007; Jessen *et al*., 2020). For example, herbivores can prefer plants with fast‐growing traits due to their lower anti‐herbivore defense and higher palatability (Eskelinen *et al*., 2012; Kaarlejarvi *et al*., 2017), or due to their greater size, as taller plants are more visible and accessible to herbivores (Díaz *et al*., 2001).

Although all these leaf economics and size‐related traits can be important in mediating the effects of nutrients and herbivores on plant biomass, their relative importance may not be constant in different environments. Size‐related traits (e.g. plant height) may be more important than leaf economics spectrum traits for plant biomass production in nutrient‐rich environments because plant growth is more limited by light than by nutrients, and taller stature should benefit plants more under such conditions (Tilman, 1988; Xiao *et al*., 2021). However, only a few studies have distinguished the relative importance of leaf‐economics vs size‐related traits in driving plant biomass response to experimental nutrient addition and herbivory exclusion across different environments.

Changes in community functional trait composition can result from both species turnover and within‐species trait changes (i.e. intraspecific trait variation) (Lepš *et al*., 2011; Siefert *et al.*, 2015; Bjorkman *et al*., 2018; Zhou *et al*., 2018, 2024; Wang *et al*., 2022). Most studies investigating nutrient and herbivore effects on plant communities have only focused on between‐species trait differences, related to species turnover, but ignore intraspecific trait variation. However, intraspecific trait variation can be an equally or more important modulator of nutrient and consumer effects on plant community composition and functioning, especially in communities with low species richness and in communities dominated by species with broad niche width or high phenotypic plasticity (Firn *et al*., 2019; Jessen *et al*., 2020; Jónsdóttir *et al*., 2022; Pichon *et al*., 2022; Zheng *et al*., 2022). Due to phenotypic plasticity, nutrient addition can alter traits of plant individuals, for example, by increasing SLA, leaf nitrogen concentration, and height (Pichon *et al*., 2022; Wang *et al*., 2022; Zheng *et al*., 2022). These intraspecific trait changes may also lead to increased accessibility and palatability of plants to herbivores (Lind *et al*., 2013; Jessen *et al*., 2020). However, only a few studies have investigated the relative contribution of intra‐ vs interspecific trait variation to changes in community‐level functioning (i.e. plant biomass production) in response to nutrient addition and herbivory (e.g. Zhou *et al*., 2018, 2024; Zheng *et al*., 2022; Wang *et al*., 2023).

Here, we report results from a full‐factorial, long‐term field experiment where we manipulated soil nutrients and herbivore presence with four treatments (control, fence, nutrient, nutrient + fence) in two grasslands differing in initial productivity. We measured six functional traits related to plant responses to nutrients and herbivores, either through leaf economics spectrum traits (SLA, LDMC, leaf C : N), plant size traits (leaf area, height), or plant defense traits (leaf tannin concentration), and tested how nutrients and herbivores influence plant community biomass through altering intra‐ and interspecific trait variations. We hypothesized that (1) nutrient addition would shift leaf economics traits (including species turnover and intraspecific traits) from slow‐ to fast‐growing traits (i.e. increasing SLA and decreasing LDMC and leaf C : N) and would also increase size‐related traits (i.e. plant height and leaf area); (2) herbivory would have opposite effects on these traits and would also increase leaf tannin concentrations, which are related to increased defense against herbivory. We further distinguished the relative contributions of trait‐based species turnover and intraspecific trait variation to the changes in plant community functional composition and total biomass under nutrient addition and herbivore exclusion. Given the low temperature, short growing season, and long‐lived species in the study area that can slow species turnover (Happonen *et al*., 2019), we predicted that (3) intraspecific trait change would be more important than trait‐based species turnover in predicting variation in plant community functional composition and aboveground biomass. The inclusion of two grasslands differing in their initial productivity and species composition allowed us to evaluate the consistency of trait responses to the treatments and how traits are linked to variations in biomass production.

## Materials and Methods

### Study area

Our study was conducted in Kilpisjärvi, northwestern Finland. The mean annual temperature in this area is −1.9°C, and the mean annual precipitation is 487 mm (Pirinen *et al*., 2012). The study area is characterized by high‐latitude mountain landscapes where lower elevations (< 650 m above sea level (asl)) are characterized by mountain birch forests and higher‐productivity grasslands, and higher elevations (> 650 m asl) by a treeless tundra zone with heaths and lower‐productivity grasslands. The main herbivores in the study area are reindeer (*Rangifer tarandus* L.), Norway lemming (*Lemmus lemmus* L.), and voles (mainly *Myodes rufocanus* Sundevall, 1846), and the grasslands are the main grazing ranges for reindeer in summer.

The experiment was conducted at two grassland sites differing in elevation and environmental and climatic conditions. The first site is located on a modest NW‐facing slope above the treeline (69°03′24″N, 20°52′24″E, 730 m asl). At this site, vegetation is dominated by small graminoids (e.g. *Agrostis mertensii* Trin., *Anthoxanthum nipponicum* L., *Carex lachenalii* Schkuhr., and *Festuca ovina* L.), low herbs (*Antennaria dioica* (L.) Gaertn., *Sibbaldia procumbens* L., *Solidago virgaurea* L., and *Veronica alpina* L.), and creeping arctic–alpine dwarf shrubs (*Cassiope hypnoides* L. and *Salix herbacea* L.). The second grassland site is located on a modest slope below the treeline (69°02′12″N, 20°50′ 6″E, 600 m asl) formed by mountain birch (*Betula pubescens* ssp. *czerepanovii* (N.I. Orlova) Hämet‐Ahti); vegetation is dominated by relatively tall forbs and legumes (e.g. *Alchemilla glomerulans* L., *Anthriscus sylvestris* (L.) Hoffm., *Astragalus frigidus* (L.) A. Gray, *Geranium sylvaticum* L., *Saussurea alpina* (L.) DC., and *Trollius europaeus* L.), and graminoids (e.g. *Elymus mutabilis* (Drobow) Tzvelev, *Milium effusum* L., and *Poa alpigena* (Blytt) Lindm.). In general, the second grassland is more productive than the first grassland site (peak aboveground biomass 387 g m^−2^ vs 251 g m^−2^) due to relatively high soil pH (6.1 vs 4.6), calcareous bedrock and relatively favorable temperature (mean temperature in the growing season 12°C vs 11°C) and snow‐free period (June 1 to October 15 vs June 12 to October 15) due to the south–west orientation of the slope and lower elevation. Given these differences in site conditions, species composition, and productivity, we refer to these two sites as ‘low‐productivity grassland’ and ‘high‐productivity grassland’.

### Experimental design

The experimental design follows the protocol of the globally distributed Nutrient Network experiment (Borer *et al*., 2014a). We applied a full factorial combination of nutrient addition and mammalian herbivore exclusion via fencing (control, fence, nutrient, nutrient + fence) to 5 m × 5 m plots at the two sites, that is, low‐productivity grassland and high‐productivity grassland. The treatments were randomly arranged within blocks that were replicated four times in each grassland, resulting in 16 plots per site. For the herbivore exclusion treatment, we caged the entire plot with a 130 cm tall fence to exclude large mammalian herbivores (e.g. reindeer) and with an 80 cm tall metal net (mesh size 1 cm), 30 cm outward‐facing flange stapled to the ground to exclude small mammalian herbivores (e.g. Norway lemming, voles). We fertilized the nutrient addition plots with 10 g N m^−2^ as time‐release urea, 10 g P m^−2^ as triple‐super phosphate, and 10 g K m^−2^ as potassium sulfate annually. During the first experimental year, we also applied a micronutrient mix (Fe, S, Mg, Mn, Cu, Zn, B, and Mo) with 100 g m^−2^ to the nutrient addition plots. We started the nutrient addition and herbivore exclusion treatments in 2014 in the low‐productivity grassland and from 2015 in the high‐productivity grassland.

### Measurements

#### Plant community survey

In early August 2022, we visually estimated the percentage cover of all vascular plant species in one permanent 1 × 1 m quadrat per plot. To measure biomass production, we clipped the total aboveground biomass (live and dead biomass) of all vascular plants rooted within two 0.1 × 1 m strips in each experimental plot next to the 1 × 1 m permanent quadrat. The clipped vegetation was dried at 60°C for 48 h, and weighed to the nearest 0.01 g. To calculate light penetration (the proportion of transmitted light through the canopy to the ground surface), we also measured the photosynthetically active radiation (PAR) above the plant canopy and at ground level in each quadrat.

#### Functional trait measurements

We measured six functional traits related to plant growth, competition, and defense. Across these traits, there were three traits related to leaf economics spectrum (SLA, LDMC, and leaf C : N), two traits related to plant size (height and leaf area), and one trait related to plant defense (leaf tannin concentration). In each plot, we selected species that accounted for > 80% of the relative vegetative cover and measured their functional traits following standard protocols (Cornelissen *et al*., 2003; Perez‐Harguindeguy *et al*., 2013) at the end of July 2022 (i.e. at the peak vegetation biomass). For each selected species in each plot, we measured plant maximum height from five fully developed and undamaged individuals in the field. We also randomly sampled five healthy, mature, and fully expanded leaves for each selected species from each plot to measure leaf area, SLA, and LDMC. SLA was calculated as leaf area per unit of dry leaf mass, and LDMC was calculated as dry leaf mass per unit of fresh leaf mass. We also collected enough leaf material from at least five individuals of each species in each plot for leaf C : N and tannin concentration analyses. The leaf C and N content were analyzed by using a CHNS/O analyzer (Thermo Fisher Scientific, Waltham, MA, USA). For the leaf tannin concentration analysis, leaf samples were freeze‐dried, ground to a powder, and analyzed using the HCl–butanol assay (Shay *et al*., 2017).

### Data analyses

We used R statistical software (R v.4.4.0) to perform all statistical analyses (R Core Team, 2024). All linear mixed‐effects models below were fitted by using the ‘lme’ function in the nlme package (Pinheiro *et al*., 2024). *F*‐statistics and associated *P*‐values for the fixed effects were obtained with the ‘anova.lme’ function in the nlme package, which applies the Satterthwaite estimate (Pinheiro *et al*., 2024). We tested for normality of residuals and homoskedasticity using graphical model validation, and log‐transformed the data when necessary.

#### Nutrient and herbivore effects on functional traits

We calculated community weighted means (CWM) of each measured functional trait based on the percentage cover of each selected plant species and its trait value in each plot. Specifically, we used the ‘trait_np_bootstrp’ function in the traitstrap package by bootstrapping trait values proportional to species percentage cover in each plot to calculate the CWM traits (Maitner *et al*., 2023; Telford *et al*., 2023). Traits were resampled 100 times, and the resulting means were averaged to obtain the final CWM of each trait. This CWM trait value represented the total trait variation in each plot, including variation caused by species turnover and intraspecific traits.

Then, to test how nutrient addition, herbivore exclusion, and their interaction affect CWM of each functional trait at each site, we fitted a set of linear mixed‐effects models separately for each trait at each site. In each model, nutrient addition, herbivore exclusion, and their interaction were the fixed effects, and block was the random effect.

Further, to assess the effects of nutrient addition, herbivore exclusion and their interaction on community trait composition at each site, we applied permutational analysis of variance (PERMANOVA; ‘adonis2’ function in the vegan package, Oksanen *et al*., 2024) with Bray–Curtis dissimilarity measure (‘vegdist’ function in the vegan package) and 999 permutations based on the six measured traits. We used nonmetric multidimensional scaling (NMDS; ‘metaMDS’ function in the vegan package) with Bray–Curtis dissimilarity to illustrate treatment and habitat effects on plant community functional trait composition visually.

#### Relative contributions of inter‐ and intraspecific trait variation

As changes in community‐level traits (CWM) can be caused by both trait‐based species turnover and intraspecific trait variation, we tested how these two components responded to treatments and further quantified their relative contributions to the changes in CWM of each trait through the sum of square decomposition (Lepš *et al*., 2011).

First, we calculated the community weighted fixed mean (CWM_fixed_), which reflected changes in CWM of traits caused by species turnover alone. Specifically, we calculated the mean trait value for each species across all control plots (or across ambient conditions if a species lacked trait data in control plots) and used the ‘trait_np_bootstrp’ function in the traitstrap package by bootstrapping these mean trait values proportional to species percentage cover in each plot to calculate the CWM_fixed_ traits (Maitner *et al*., 2023; Telford *et al*., 2023). Traits were resampled 100 times, and the resulting means were averaged to obtain the final CWM_fixed_ of each trait. Differences between CWM (see above) and CWM_fixed_ reflect changes in CWM of traits caused by intraspecific trait variation (ITV).

Second, to test how nutrient addition, herbivore exclusion, and their interaction drive trait changes induced by species turnover or intraspecific trait variation, we fitted separate linear mixed‐effects models for each trait at each site, using either CWM_fixed_ or ITV as the response variable, nutrient addition, herbivore exclusion, and their interactions as fixed effects, and block as the random effect.

Third, we used the sum of squares decomposition to quantify the relative contribution of species turnover and intraspecific trait variation to changes in CWM of traits across all treatments at each site. Specifically, we ran separate ANOVAs on CWM, CWM_fixed_, or ITV of each trait for each site with nutrient addition, herbivore exclusion and their interaction as predictors and then decomposed the sum of squares from these ANOVAs to quantify the relative contribution of species turnover and intraspecific trait variation (Lepš *et al*., 2011).

#### Relationships between functional traits and aboveground biomass of plant communities

To assess the effects of nutrient addition, herbivore exclusion, and their interaction on aboveground biomass of plant communities at the two sites, we fitted two linear mixed‐effects models with nutrient addition, herbivore exclusion, and their interactions as fixed effects, and block as the random effect.

To explore the relationship between plant functional traits and aboveground biomass, we also fitted linear mixed‐effects models to test the relationships between aboveground biomass and CWM of each measured trait across all treatments at each site. In these models, plant community biomass was the response variable and CWM of each trait was the fixed effect, each in its own model, and block was treated as the random effect. To further investigate whether species turnover or intraspecific trait variation drives these relationships, we conducted similar linear mixed‐effects models to examine the relationships between CWM_fixed_ or ITV of each trait and aboveground biomass for each site. Marginal and conditional r squares for each model were calculated using ‘rsquare’ function in the piecewisesem package (Lefcheck, 2016).

## Results

### Nutrient and herbivore effects on functional traits

**Fig. 1:**
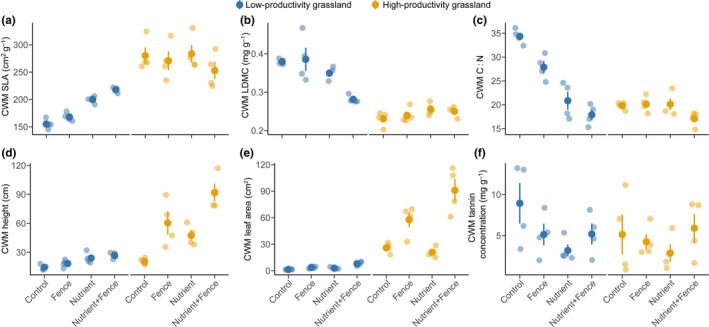
Responses of community weighted mean (CWM) traits to nutrient addition and herbivore exclusion in the low‐productivity and high‐productivity grassland. (a) CWM specific leaf area (SLA), (b) leaf dry matter content (LDMC), and (c) foliar C : N are plant economics spectrum traits; (d) CWM plant height and (e) leaf area are plant size traits; (f) CWM tannin concentration is an indicator of plant defense ability. Dots with deep colors represent the mean CWM values of each treatment, and points with light colors represent the raw CWM values of each plot. Error bars represent ±SE.

The CWM traits showed distinct responses to nutrient addition and herbivore exclusion in the low‐productivity and high‐productivity grassland. Nutrient addition increased CWM SLA (Nutrient addition: *P* < 0.01; Fig. 1a; Supporting Information Table S1) and leaf area (Nutrient addition: *P* < 0.01; Fig. 1e; Table S1), but decreased CWM LDMC (Nutrient addition: *P* < 0.01; Fig. 1b; Table S1) and leaf C : N (Nutrient addition: *P* < 0.01; Fig. 1c; Table S1) in the low‐productivity grassland. Nutrient addition also increased CWM height both in the low‐productivity (Nutrient addition: *P* < 0.01; Fig. 1e; Table S1) and high‐productivity grassland (Nutrient addition: *P* < 0.01; Fig. 1e; Table S1). Similarly, herbivore exclusion increased CWM SLA (Nutrient addition: *P* < 0.01; Fig. 1a; Table S1) and decreased CWM C : N (Nutrient addition: *P* = 0.01; Fig. 1c; Table S1) in the low‐productivity grassland and increased CWM height in the high‐productivity grassland (Herbivore exclusion: *P* < 0.01; Fig. 1d; Table S1) but not in the low‐productivity grassland (Herbivore exclusion: *P* = 0.06; Fig. 1d; Table S1). Further, herbivore exclusion increased CWM leaf area both in the low‐productivity and high‐productivity grasslands (Herbivore exclusion: *P* < 0.01; Fig. 1e; Table S1), and this impact was also greater when combined with nutrient addition in the high‐productivity grassland (Nutrient × Herbivore exclusion: *P* = 0.04, Fig. 1e; Table S1). Finally, nutrient addition and herbivore exclusion did not have a significant effect on CWM leaf tannin concentration in the low‐productivity (Nutrient addition: *P* = 0.06; Herbivore exclusion: *P* = 0.51, Fig. 1f; Table S1) or the high‐productivity grassland (Nutrient addition: *P* = 0.85; Herbivore exclusion: *P* = 0.49, Fig. 1f; Table S1).

As indicated by PERMANOVA analysis and NMDS plots, the high‐productivity grassland was characterized by fast‐growing leaf economics traits and larger size, whereas the low‐productivity grassland occupied a slow‐growing part of the ordination space, characterized also by smaller size (Fig. S1; Table S2). Importantly, both nutrient addition and herbivore exclusion changed plant community trait composition (measured as Bray–Curtis dissimilarity) (Fig. S1; Table S2). Specifically, nutrient addition shifted the plant community towards faster growth (higher CWM SLA, lower CWM LDMC, and C : N) and larger size (higher CWM height and leaf area) in the low‐productivity grassland (Nutrient addition: *P* < 0.01; Fig. S1; Table S2), but this effect was marginal in the high‐productivity grassland (Nutrient addition: *P* = 0.06; Fig. S1; Table S2). Herbivore exclusion shifted the plant community towards larger size (higher CWM height and leaf area) in both low‐productivity (Herbivore exclusion: *P* < 0.01; Fig. S1a; Table S2) and high‐productivity grassland (Herbivore exclusion: *P* < 0.01; Fig. S1b; Table S2), while in the low‐productivity grassland, herbivory exclusion was also associated with increased leaf area and specific leaf area (Herbivore exclusion: *P* < 0.01; Fig. S1a; Table S2).

### Relative contributions of inter‐ and intraspecific trait variation

**Fig. 2:**
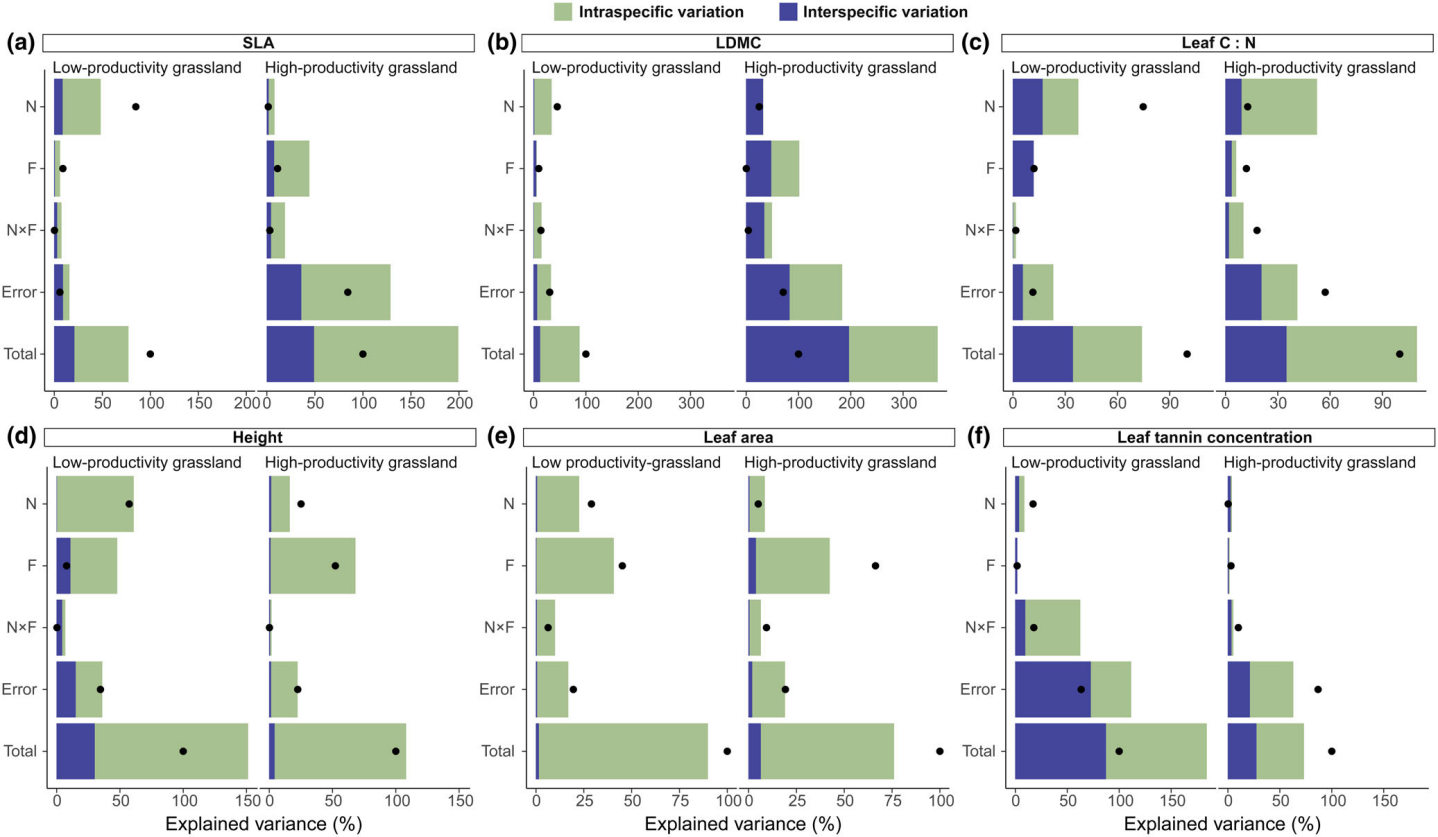
Sum of squares decomposition results showing the relative contributions of inter‐ and intraspecific trait variation to changes in community weighted mean trait values in response to nutrient addition (N), fence (F), their interactions (N × F), residual variation (Error), and total variation (Total) in the low‐productivity and high‐productivity grasslands. Bar colors indicate proportions of variation that are attributable to intraspecific variation or species turnover. Dots indicate the total variation explained by nutrient, fence, their interactions, and residual. If the total variation is greater than the sum of the two sources of variation, it suggests that there is positive covariation between the sources of variation. Likewise, the total variation is less than the sum of the two sources of variation if there is a negative covariation between the sources of variation. (a) specific leaf area, (b) leaf dry matter content (LDMC), and (c) foliar C : N are plant economics spectrum traits; (d) plant height and (e) leaf area are plant size traits; (f) tannin concentration is an indicator of plant defense ability.

Nutrient addition increased CWM_fixed_ (Nutrient addition: *P* = 0.01; Fig. S2a; Table S3) and ITV (Nutrient addition: *P* < 0.01; Fig. S3a; Table S4) of SLA, with a larger contribution of intraspecific trait variation than species turnover to changes in CWM SLA in the low‐productivity grassland (Fig. 2a). However, in the high‐productivity grassland, nutrient addition did not similarly alter species turnover and intraspecific trait variation of SLA (Figs S2a, S3a; Tables S3, S4). For other leaf economics traits, nutrient effects on CWM LDMC were also largely explained by intraspecific trait variation (Fig. 2b,c) as it increased the ITV of LDMC (Nutrient addition: *P* < 0.01; Fig. S3b; Table S4) in the low‐productivity grassland. The impact of nutrient addition on CWM C : N was approximately equally explained by intraspecific and interspecific trait variation (Fig. 2c) as nutrient addition decreased CWM_fixed_ (Nutrient addition: *P* < 0.01; Fig. S3c; Table S3) and ITV of C : N (Nutrient addition: *P* < 0.01; Fig. S3c; Table S4) in the low‐productivity grassland. Moreover, nutrient addition increased size‐related traits also through changes in intraspecific trait variation (Fig. 2d,e). Specifically, nutrient addition increased ITV of height and leaf area both in the low‐productivity (Nutrient addition: *P* < 0.01 and *P* < 0.01, respectively; Fig. S3d,e; Table S4) and high‐productivity grassland (Nutrient addition: *P* = 0.02, and *P* = 0.03, respectively; Fig. S3d,e; Table S4). Furthermore, nutrient addition decreased ITV of leaf tannin concentration in grazed plots in the low‐productivity grassland (Nutrient addition × Herbivore exclusion: *P* < 0.01; Fig. S3f; Table S4).

Herbivore exclusion increased ITV of SLA in the low‐productivity (Herbivore exclusion: *P* < 0.01, Fig. S3a; Table S4) and marginally increased it in the high‐productivity grassland (Herbivore exclusion: *P* = 0.07; Fig. S3a; Table S4), leading to a greater contribution of intraspecific trait variation than species turnover to community SLA (Fig. 2a). For the size‐related traits, the contribution of intraspecific variation was higher than species turnover to changes in CWM values in response to herbivore exclusion (Fig. 2d). Herbivore exclusion increased ITV of plant height both in the low‐productivity (Herbivore exclusion: *P* < 0.01; Fig. S3d; Table S4) and high‐productivity grassland (Herbivore exclusion: *P* < 0.01, Fig. S3d; Table S4). Similarly, herbivore effect on CWM leaf area was also largely explained by intraspecific trait variation (Fig. 2e) as it increased ITV of leaf area both in the low‐productivity (Herbivore exclusion: *P* < 0.01; Fig. S3e; Table S4) and high‐productivity grassland (Herbivore exclusion: *P* < 0.01; Fig. S3e; Table S4). Herbivore exclusion did not alter CWM_fixed_ (Herbivore exclusion: *P* = 0.46 and *P* = 0.55, in the low‐productivity and high‐productivity grassland, respectively; Fig. S2f; Table S3) and ITV of leaf tannin concentrations (Herbivore exclusion: *P* = 0.99 and *P* = 0.54, in the low‐productivity and high‐productivity grassland, respectively; Fig. S3f; Table S4).

### Relationships between functional traits and community biomass

**Fig. 3:**
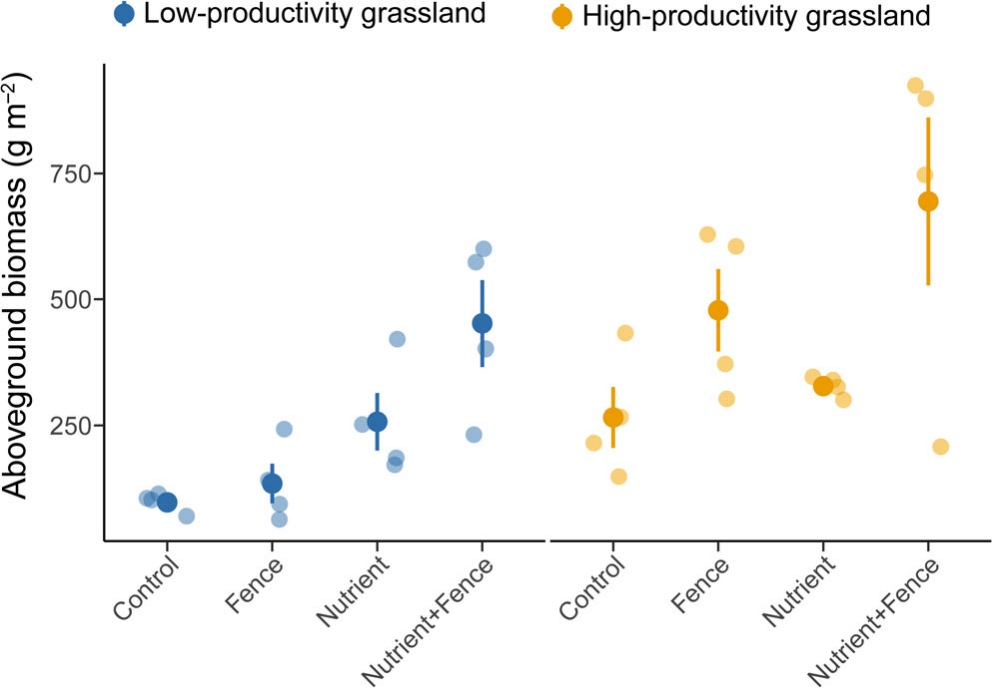
Response of total aboveground biomass (the sum of live and litter biomass) to nutrient addition and herbivore exclusion by fence in the low‐productivity and high‐productivity grassland. Dots with deep colors represent the mean community weighted mean (CWM) values of each treatment, and points with light colors represent the raw CWM values of each plot. Error bars represent ±SE.

Nutrient addition increased total aboveground biomass only in the low‐productivity grassland (Nutrient addition: *P* < 0.01; Fig. 3; Table S5), suggesting greater overall nutrient limitation of biomass production in this habitat. Herbivore exclusion only tended to increase total biomass in the low‐productivity grassland (Herbivore exclusion: *P* = 0.06; Fig. 3; Table S5) and increased it in the high‐productivity grassland (Herbivore exclusion: *P* = 0.03; Fig. 3; Table S5).

**Fig. 4:**
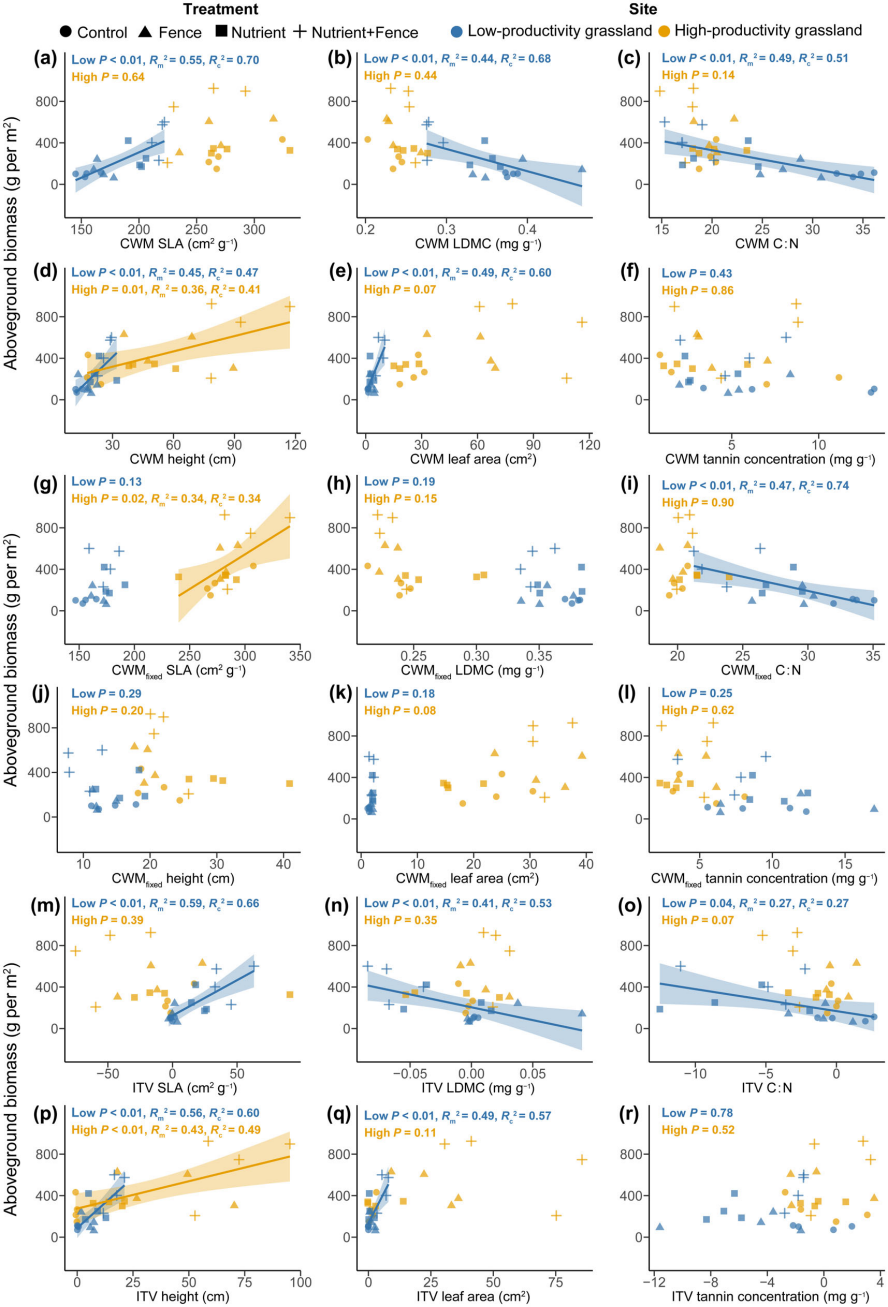
Relationships between (a–f) CWM, (g–l) CWM_fixed_, and (m–r) intraspecific trait variation (ITV) of each trait and total aboveground biomass of plant communities across all treatments in the low‐productivity and high‐productivity grassland. Only significant relationships (*P* < 0.05) are shown. Shaded areas represent 95% confidence intervals. CWM, community weighted mean.

Total aboveground biomass across all plots was related to specific functional traits in the low‐productivity and high‐productivity grassland (Fig. 4). For leaf economic traits, CWM SLA was positively related, but CWM LDMC and leaf C : N were negatively related to the total biomass in the low‐productivity grassland (Fig. 4a,c), suggesting that fast‐growing traits are linked to greater plant biomass in the nutrient‐poor tundra grassland. Both size‐related traits (i.e. CWM height and leaf area) were positively related to total biomass in both habitats (Fig. 4d,e), showing that size‐related traits are associated with plant biomass production independent of original habitat productivity. Further, the plant defense trait, CWM tannin concentration, was not significantly related to total biomass (Fig. 4f).

When we further distinguished the roles of species turnover and intraspecific trait variation in affecting biomass production, we found that total aboveground biomass was positively related to ITV of SLA and LDMC in the low‐productivity grassland (Fig. 4g), which suggests that intraspecific trait variation rather than species turnover contributes to the positive relationship between CWM SLA or CWM LDMC and biomass. However, for the leaf C : N, total aboveground biomass was negatively related to ITV but not to CWM_fixed_ in the low‐productivity grassland. We found that intraspecific trait changes drove changes in size‐related traits: both ITV of height (Fig. 4p) and leaf area (Fig. 4q) were positively related to total biomass in the low‐productivity grassland, and ITV of height was positively related to biomass in the high‐productivity grassland (Fig. 4p). Species turnover in size‐related traits was not related to biomass, either in the low‐productivity or high‐productivity grassland (Fig. 4j,k). However, total biomass was not related to CMW_fixed_ (Fig. 4l) or ITV (Fig. 4r) of tannin concentration.

## Discussion

We examined how nutrient addition and herbivore exclusion affected inter‐ and intraspecific trait composition and how these changes translated to plant biomass in two grasslands that differed in initial productivity. We found that nutrient addition and herbivore exclusion affected community functional composition in opposite ways and through distinct trait dimensions. In general, nutrient addition promoted faster growth (in terms of leaf economics traits) and greater height, and these effects were mainly driven by intraspecific trait changes. By contrast, herbivore exclusion promoted greater height and leaf area (size‐related traits) but had no impact on leaf economics traits, and these effects were, to a greater extent, driven by intraspecific trait changes. These effects also depended on the habitat, as leaf economics trait changes were generally more important in the low‐productivity grassland, while size‐related trait changes were generally more important in the high‐productivity grassland. We also found that, in general, intraspecific variation, both in terms of leaf economics and size‐related traits, contributed more to biomass change under nutrient addition and herbivore exclusion than trait‐based species turnover. Overall, these results suggest that nutrients and herbivores influence plant functional composition and total biomass through different trait‐mediated pathways, and these effects are primarily driven by intraspecific variation, with the relative importance of these mechanisms being strongly habitat‐dependent.

### Nutrient and herbivore effects on functional traits

Nutrient addition promoted fast‐growing strategies in the low‐productivity grassland, while no significant change in these leaf economic traits was observed in the high‐productivity grassland. This finding is in line with some previous work stating that plant responses to soil resource availability should be determined by traits involved in biogeochemical cycling (Lavorel & Garnier, 2002; Garnier *et al*., 2007) and that the responses of leaf economic traits to nutrients depend on habitat conditions and plant height (Cheng *et al*., 2023). Greater responsiveness of leaf economic traits in the low‐productivity grassland could be due to the prevalence of low‐statured species in this habitat that may exhibit relatively greater potential to adjust leaf economic traits in response to enhanced soil nutrients than tall plants dominant in the high‐productivity grassland (Cheng *et al*., 2023). By contrast, nutrient addition consistently increased plant height both in low‐ and high‐productivity grassland, matching some previous studies (e.g. Tatarko & Knops, 2018; Liao *et al*., 2024), and supporting a general trait‐based assembly assertion that increased nutrient availability favors tall plants superior in competition for light (Hautier *et al*., 2009; DeMalach *et al*., 2017; Xiao *et al*., 2021; Eskelinen *et al*., 2022; de Bello *et al*., 2025).

Long‐term herbivore exclusion increased plant height and leaf area particularly strongly in the high‐productivity grassland, and leaf area in the low‐productivity grassland. These results demonstrate that herbivory can be a key factor reducing the dominance of tall plants with large leaves, especially in more productive grassland habitats with relatively intense grazing pressure. Our finding aligns with previous studies showing that herbivory can have strong impacts on plant communities through alleviating competition for light (Borer *et al*., 2014b; Eskelinen *et al*., 2022) and further suggests that these impacts on plant communities and competition can be translated through shifts in size‐related plant trait dimensions. In contrast to size‐related traits, herbivore exclusion had a significant effect on leaf economic spectrum traits only in the low‐productivity grassland, where herbivore exclusion increased SLA and decreased C : N. In line with some other studies (Díaz *et al*., 2007; Jessen *et al*., 2020; Jiang *et al*., 2023), these results suggest that, under grazing, resource‐conservative and nutrient‐poor plants are favored, at least in low‐productivity conditions. However, CWM tannin showed only a weak response to herbivore exclusion, although we could have expected herbivore exclusion to lead to a decline in well‐defended plants (Carmona *et al*., 2011; Speed *et al*., 2014). In general, tannins may play a greater role in defense against insect herbivores (War *et al*., 2012), and high variation in CWM tannin concentrations among our experimental plots may limit finding a possible response (Salminen & Karonen, 2011). Previous consumer‐resource studies suggest that herbivory can counteract nutrient effects on plant communities (Worm *et al*., 2002; Hillebrand *et al*., 2007; Anderson *et al*., 2018), but full‐factorial experimental tests on such interactive effects on plant functional traits are largely missing, which hampers developing a better understanding of the role of trophic interactions in the context of trait‐based ecology (de Bello *et al*., 2025). In our experiment, nutrients and herbivory mostly acted independently and influenced distinct trait dimensions: nutrients predominantly shaped leaf economics and size traits in the low‐productivity grassland, while herbivores influenced plant size in the high‐productivity grassland. However, we also found a few interactions. For example, herbivore exclusion and nutrients jointly caused the greatest decline in CWM LDMC in the low‐productivity grassland. Further, in the high‐productivity grassland, nutrient addition and herbivore exclusion synergized to increase CWM leaf area. These examples demonstrate that nutrients and reduced herbivory can also amplify each other's effects. Taken together, our findings underscore the need to consider how different global change drivers may affect plant communities through distinct trait dimensions and the complexity of trait responses to the interactive effects of multiple global change drivers (Harley *et al*., 2006; Bruelheide *et al*., 2018; Henn *et al*., 2024; de Bello *et al*., 2025).

### Relative contributions of inter‐ and intraspecific trait variation

Disentangling the contributions of species turnover and intraspecific trait change revealed distinct mechanisms underlying trait responses (Lepš *et al*., 2011). In the low‐productivity grassland, nutrient‐driven shifts in SLA were mainly driven by intraspecific trait variation, suggesting that trait plasticity drives shifts in resource‐use strategies under changing nutrient availability (Albert *et al*., 2010; Lepš *et al*., 2011). This finding is consistent with previous studies reporting that phenotypic plasticity is an essential pathway for community‐level trait variation in harsh environments (Pérez‐Ramos *et al*., 2019; Jónsdóttir *et al*., 2022). In addition, the nutrient‐induced increase in plant height in both grasslands was primarily driven by intraspecific trait change. This finding, in line with some other studies (Siefert & Ritchie, 2016; Zhang *et al*., 2022), suggests that species already present in the communities respond plastically to nutrient addition by increasing height, rather than being replaced by inherently taller species. Intraspecific trait plasticity in height likely confers a competitive advantage under nutrient‐induced light limitation, allowing individuals to grow taller than others (Funk *et al*., 2017; Carmona *et al*., 2019).

Herbivore exclusion similarly drove an increase in plant height and leaf area primarily through intraspecific trait variation, particularly in the high‐productivity grassland. The restricted response of interspecific turnover to herbivore exclusion highlights that plasticity in size‐related traits is a central mechanism through which communities can adjust to altered biotic pressures (Mitchell & Bakker, 2016; Jessen *et al*., 2020). This plastic response may allow plant individuals to take advantage of reduced herbivory by allocating resources to grow larger and taller, thereby enhancing light acquisition in denser canopies (Boege, 2010; Schiestl, 2014). Our findings thus show that intraspecific height responses can be important mediators of community responses to nutrient addition and herbivory, while interspecific height differences may have limited predictive power (Eskelinen *et al*., 2022). Overall, our results demonstrate that intraspecific trait variation can be the dominant source of functional change in response to nutrient enrichment and changes in grazing pressure, particularly for traits associated with size and light competition.

### Relationships between functional traits and total biomass

Understanding how trait changes scale to ecosystem functioning is critical for predicting ecosystem responses to global change (Funk *et al*., 2017; Hagan *et al*., 2023; de Bello *et al*., 2025). We found that functional trait–biomass relationships differed between the two grasslands, reflecting contrasting environmental limitations. In the low‐productivity grassland, total aboveground biomass was positively associated with both SLA and C : N as well as with plant height, indicating that both resource acquisition and size traits contribute to productivity in resource‐limited environments. These patterns suggest that nutrient addition facilitates biomass accumulation via both enhancing resource acquisition and light capture, supporting previous work on trait–function linkages (Laughlin, 2014; Reich, 2014). In the high‐productivity grassland, however, only height was significantly related to total biomass, suggesting that once nutrients and herbivory are no longer limiting, light becomes the primary limiting resource for plant growth. This habitat productivity‐dependent role of traits in mediating biomass responses emphasizes the importance of identifying the dominant limiting factor in each system.

Importantly, these trait–biomass relationships were primarily driven by intraspecific variation rather than interspecific turnover. For example, strong intraspecific changes in SLA, LDMC, and leaf C : N under nutrient addition indicate considerable morphological and physiological plasticity that enhances growth potential, even in these grasslands with a cold climate and high latitude (but see Stotz *et al*., 2021). Similarly, the increase in plant height driven by both nutrient enrichment and herbivore exclusion suggests that intraspecific trait variation in height benefits community biomass production. These findings reinforce recent calls to incorporate intraspecific traits into models linking traits to ecosystem processes (Violle *et al*., 2012; Funk *et al*., 2017; Jónsdóttir *et al*., 2022). In systems undergoing rapid environmental change, trait plasticity may provide a critical buffer that enables continued ecosystem function.

Our study demonstrates that nutrient addition and herbivore exclusion shape grassland plant communities through distinct trait pathways, ultimately driving differences in community composition and ecosystem productivity. Nutrient addition primarily altered leaf economics traits both through inducing species turnover and intraspecific variation, especially in the low‐productivity system, while herbivore exclusion mainly influenced size‐related traits through intraspecific variation, particularly in the high‐productivity environment. Moreover, intraspecific variation, both in terms of leaf economics and size‐related traits, contributed more to biomass change under nutrient addition and herbivore exclusion than trait‐based species turnover. Together, our findings reinforce the value of a trait‐based approach in understanding how multiple global change drivers – acting through different trait dimensions – shape plant communities and their functional outcomes across contrasting environmental contexts. Recognizing the differential roles of intra‐ and interspecific trait variation across trait dimensions and environments is essential for predicting how functional composition and ecosystem processes of grasslands will respond to global changes.

## Competing interests

None declared.

## Author contributions

XY, RV and AE designed the research. XY, AE and RV conducted the field experiments. XY performed the lab analyses. XY, RV and AE analyzed the data. XY wrote the first draft and all authors contributed to reviewing, editing and revising the manuscript.

## Disclaimer

The New Phytologist Foundation remains neutral with regard to jurisdictional claims in maps and in any institutional affiliations.

## Supporting information


**Fig. S1** Plant community trait composition of plots across all treatments in the low‐productivity and high‐productivity grassland by nonmetric multidimensional scaling analysis, in two‐dimensional space.
**Fig. S2** Responses of community‐level trait variations induced by interspecific trait variations to nutrient addition and herbivore exclusion by fence in the low‐productivity and the high‐productivity grassland.
**Fig. S3** Responses of community‐level trait variations induced by intraspecific trait variations to nutrient addition and herbivore exclusion by fence in the low‐productivity and the high‐productivity grassland.
**Table S1** Results of the linear mixed‐effects models testing effects of nutrient addition and herbivore exclusion by fence on community weight mean of functional traits for the low‐productivity and the high‐productivity grassland separately.
**Table S2** Results of the permutation test of the effects of nutrient addition and herbivore exclusion by fence on plant community trait composition for the low‐productivity and the high‐productivity grassland separately.
**Table S3** Results of the linear mixed‐effects models testing effects of nutrient addition and herbivore exclusion by fence on community‐level trait variations induced by interspecific variations for the low‐productivity and the high‐productivity grassland separately.
**Table S4** Results of the linear mixed‐effects models testing effects of nutrient addition and herbivore exclusion by fence on community‐level trait variations induced by intraspecific variations for the low‐productivity and the high‐productivity grassland separately.
**Table S5** Results of the linear mixed‐effects models testing effects of nutrient addition and herbivore exclusion by fence on total community biomass for the low‐productivity and the high‐productivity grassland separately.
Please note: Wiley is not responsible for the content or functionality of any Supporting Information supplied by the authors. Any queries (other than missing material) should be directed to the *New Phytologist* Central Office.

## Data Availability

Data are publicly available in the Figshare (doi: 10.6084/m9.figshare.30816602).

## References

[nph70827-bib-0001] Albert CH , Grassein F , Schurr FM , Vieilledent G , Violle C . 2010. When and how should intraspecific variability be considered in trait‐based plant ecology? Perspectives in Plant Ecology, Evolution and Systematics 13: 217–225.

[nph70827-bib-0002] Anderson TM , Griffith DM , Grace JB , Lind EM , Adler PB , Biederman LA , Blumenthal DM , Daleo P , Firn J , Hagenah N *et al*. 2018. Herbivory and eutrophication mediate grassland plant nutrient responses across a global climatic gradient. Ecology 99: 822–831. 29603733 10.1002/ecy.2175

[nph70827-bib-0003] Bakker ES , Ritchie ME , Olff H , Milchunas DG , Knops JMH . 2006. Herbivore impact on grassland plant diversity depends on habitat productivity and herbivore size. Ecology Letters 9: 780–788. 16796567 10.1111/j.1461-0248.2006.00925.x

[nph70827-bib-0004] de Bello F , Fischer FM , Puy J , Shipley B , Verdú M , Götzenberger L , Lavorel S , Moretti M , Wright IJ , Berg MP *et al*. 2025. Raunkiæran shortfalls: challenges and perspectives in trait‐based ecology. Ecological Monographs 95: e70018.

[nph70827-bib-0005] Bjorkman AD , Myers‐Smith IH , Elmendorf SC , Normand S , Ruger N , Beck PSA , Blach‐Overgaard A , Blok D , Cornelissen JHC , Forbes BC *et al*. 2018. Plant functional trait change across a warming tundra biome. Nature 562: 57. 30258229 10.1038/s41586-018-0563-7

[nph70827-bib-0007] Boege K . 2010. Induced responses to competition and herbivory: natural selection on multi‐trait phenotypic plasticity. Ecology 91: 2628–2637. 20957957 10.1890/09-0543.1

[nph70827-bib-0008] Borer ET , Harpole WS , Adler PB , Arnillas CA , Bugalho MN , Cadotte MW , Caldeira MC , Campana S , Dickman CR , Dickson TL *et al*. 2020. Nutrients cause grassland biomass to outpace herbivory. Nature Communications 11: 6036. 10.1038/s41467-020-19870-y PMC7695826 33247130

[nph70827-bib-0009] Borer ET , Harpole WS , Adler PB , Lind EM , Orrock JL , Seabloom EW , Smith MD . 2014a. Finding generality in ecology: a model for globally distributed experiments. Methods in Ecology and Evolution 5: 65–73.

[nph70827-bib-0010] Borer ET , Seabloom EW , Gruner DS , Harpole WS , Hillebrand H , Lind EM , Adler PB , Alberti J , Anderson TM , Bakker JD *et al*. 2014b. Herbivores and nutrients control grassland plant diversity via light limitation. Nature 508: 517–520. 24670649 10.1038/nature13144

[nph70827-bib-0011] Borer ET , Stevens CJ . 2022. Nitrogen deposition and climate: an integrated synthesis. Trends in Ecology & Evolution 37: 541–552. 35428538 10.1016/j.tree.2022.02.013

[nph70827-bib-0012] Bruelheide H , Dengler J , Purschke O , Lenoir J , Jiménez‐Alfaro B , Hennekens SM , Botta‐Dukát Z , Chytry M , Field R , Jansen F *et al*. 2018. Global trait‐environment relationships of plant communities. Nature Ecology & Evolution 2: 1906–1917. 30455437 10.1038/s41559-018-0699-8

[nph70827-bib-0013] Carmona CP , de Bello F , Azcárate FM , Mason NWH , Peco B . 2019. Trait hierarchies and intraspecific variability drive competitive interactions in Mediterranean annual plants. Journal of Ecology 107: 2078–2089.

[nph70827-bib-0014] Carmona D , Lajeunesse MJ , Johnson MTJ . 2011. Plant traits that predict resistance to herbivores. Functional Ecology 25: 358–367.

[nph70827-bib-0015] Cheng Y , Liu X , Song Z , Ma M , Zhou S , Allan E . 2023. Divergent trait responses to nitrogen addition in tall and short species. Journal of Ecology 111: 1443–1454.

[nph70827-bib-0016] Cornelissen JHC , Lavorel S , Garnier E , Díaz S , Buchmann N , Gurvich DE , Reich PB , ter Steege H , Morgan HD , van der Heijden MGA *et al*. 2003. A handbook of protocols for standardised and easy measurement of plant functional traits worldwide. Australian Journal of Botany 51: 335–380.

[nph70827-bib-0017] DeMalach N , Zaady E , Kadmon R . 2017. Light asymmetry explains the effect of nutrient enrichment on grassland diversity. Ecology Letters 20: 60–69. 27933739 10.1111/ele.12706

[nph70827-bib-0018] DeMalach N , Zaady E , Weiner J , Kadmon R . 2016. Size asymmetry of resource competition and the structure of plant communities. Journal of Ecology 104: 899–910.

[nph70827-bib-0019] Díaz S , Kattge J , Cornelissen JHC , Wright IJ , Lavorel S , Dray S , Reu B , Kleyer M , Wirth C , Prentice IC *et al*. 2016. The global spectrum of plant form and function. Nature 529: 167–171. 26700811 10.1038/nature16489

[nph70827-bib-0020] Díaz S , Lavorel S , McIntyre S , Falczuk V , Casanoves F , Milchunas DG , Skarpe C , Rusch G , Sternberg M , Noy‐Meir I *et al*. 2007. Plant trait responses to grazing: a global synthesis. Global Change Biology 13: 313–341.

[nph70827-bib-0021] Díaz S , Noy‐Meir I , Cabido M . 2001. Can grazing response of herbaceous plants be predicted from simple vegetative traits? Journal of Applied Ecology 38: 497–508.

[nph70827-bib-0081] Elser JJ , Bracken MES , Cleland EE , Gruner DS , Harpole WS , Hillebrand H , Ngai JT , Seabloom EW , Shurin JB , Smith JE . 2007. Global analysis of nitrogen and phosphorus limitation of primary producers in freshwater, marine and terrestrial ecosystems. Ecology Letters 10: 1135–1142. 17922835 10.1111/j.1461-0248.2007.01113.x

[nph70827-bib-0022] Eskelinen A , Harpole WS , Jessen MT , Virtanen R , Hautier Y . 2022. Light competition drives herbivore and nutrient effects on plant diversity. Nature 611: 301–305. 36323777 10.1038/s41586-022-05383-9 PMC9646529

[nph70827-bib-0023] Eskelinen A , Harrison S , Tuomi M . 2012. Plant traits mediate consumer and nutrient control on plant community productivity and diversity. Ecology 93: 2705–2718. 23431600 10.1890/12-0393.1

[nph70827-bib-0024] Fay PA , Prober SM , Harpole WS , Knops JMH , Bakker JD , Borer ET , Lind EM , MacDougall AS , Seabloom EW , Wragg PD *et al*. 2015. Grassland productivity limited by multiple nutrients. Nature Plants 1: 1508. 10.1038/nplants.2015.80 27250253

[nph70827-bib-0025] Firn J , Nguyen H , Schütz M , Risch AC . 2019. Leaf trait variability between and within subalpine grassland species differs depending on site conditions and herbivory. Proceedings of the Royal Society B: Biological Sciences 286: 400–406. 10.1098/rspb.2019.0429 PMC6661350 31337314

[nph70827-bib-0026] Funk JL , Larson JE , Ames GM , Butterfield BJ , Cavender‐Bares J , Firn J , Laughlin DC , Sutton‐Grier AE , Williams L , Wright J . 2017. Revisiting the Holy Grail: using plant functional traits to understand ecological processes. Biological Reviews 92: 1156–1173. 27103505 10.1111/brv.12275

[nph70827-bib-0027] Garnier E , Lavorel S , Ansquer P , Castro H , Cruz P , Dolezal J , Eriksson O , Fortunel C , Freitas H , Golodets C *et al*. 2007. Assessing the effects of land‐use change on plant traits, communities and ecosystem functioning in grasslands: a standardized methodology and lessons from an application to 11 European sites. Annals of Botany 99: 967–985. 17085470 10.1093/aob/mcl215 PMC2802906

[nph70827-bib-0028] Hagan JG , Henn JJ , Osterman WHA . 2023. Plant traits alone are good predictors of ecosystem properties when used carefully. Nature Ecology & Evolution 7: 332–334. 36646946 10.1038/s41559-022-01920-x

[nph70827-bib-0029] Happonen K , Aalto J , Kemppinen J , Niittynen P , Virkkala AM , Luoto M . 2019. Snow is an important control of plant community functional composition in oroarctic tundra. Oecologia 191: 601–608. 31522244 10.1007/s00442-019-04508-8 PMC6825026

[nph70827-bib-0030] Harley CDG , Hughes AR , Hultgren KM , Miner BG , Sorte CJB , Thornber CS , Rodriguez LF , Tomanek L , Williams SL . 2006. The impacts of climate change in coastal marine systems. Ecology Letters 9: 228–241. 16958887 10.1111/j.1461-0248.2005.00871.x

[nph70827-bib-0031] Hautier Y , Niklaus PA , Hector A . 2009. Competition for light causes plant biodiversity loss after eutrophication. Science 324: 636–638. 19407202 10.1126/science.1169640

[nph70827-bib-0032] Henn J , Anderson K , Brigham L , Bueno de Mesquita C , Collins C , Elmendorf S , Green M , Huxley J , Rafferty N , Rose‐Person A *et al*. 2024. Long‐term alpine plant responses to global change drivers depend on functional traits. Ecology Letters 27: e14518. 39412423 10.1111/ele.14518

[nph70827-bib-0033] Hillebrand H , Gruner DS , Borer ET , Bracken MES , Cleland EE , Elser JJ , Harpole WS , Ngai JT , Seabloom EW , Shurin JB *et al*. 2007. Consumer versus resource control of producer diversity depends on ecosystem type and producer community structure. Proceedings of the National Academy of Sciences, USA 104: 10904–10909. 10.1073/pnas.0701918104 PMC1904146 17581875

[nph70827-bib-0034] Huisman J , Grover JP , van der Wal R , van Andel J . 1999. Competition for light, plant‐species replacement and herbivore abundance along productivity gradients. In: Olff H , Brown VK , Drent RH , eds. Herbivores: between plants and predators. Oxford, UK: Blackwell Science.

[nph70827-bib-0035] Isbell F , Reich PB , Tilman D , Hobbie SE , Polasky S , Binder S . 2013. Nutrient enrichment, biodiversity loss, and consequent declines in ecosystem productivity. Proceedings of the National Academy of Sciences, USA 110: 11911–11916. 10.1073/pnas.1310880110 PMC3718098 23818582

[nph70827-bib-0036] Jessen MT , Kaarlejarvi E , Olofsson J , Eskelinen A . 2020. Mammalian herbivory shapes intraspecific trait responses to warmer climate and nutrient enrichment. Global Change Biology 26: 6742–6752. 33020977 10.1111/gcb.15378

[nph70827-bib-0037] Jia SH , Wang XG , Yuan ZQ , Lin F , Ye J , Hao ZQ , Luskin MS . 2018. Global signal of top‐down control of terrestrial plant communities by herbivores. Proceedings of the National Academy of Sciences, USA 115: 6237–6242. 10.1073/pnas.1707984115 PMC6004463 29848630

[nph70827-bib-0038] Jiang S , Zhang J , Tang Y , Li Z , Liu H , Wang L , Wu Y , Liang C . 2023. Plant functional traits and biodiversity can reveal the response of ecosystem functions to grazing. Science of the Total Environment 899: 165636. 37487897 10.1016/j.scitotenv.2023.165636

[nph70827-bib-0039] Jónsdóttir IS , Halbritter AH , Christiansen CT , Althuizen IHJ , Haugum SV , Henn JJ , Björnsdóttir K , Maitner BS , Malhi Y , Michaletz ST *et al*. 2022. Intraspecific trait variability is a key feature underlying high arctic plant community resistance to climate warming. Ecological Monographs 93: e1555.

[nph70827-bib-0040] Kaarlejarvi E , Eskelinen A , Olofsson J . 2017. Herbivores rescue diversity in warming tundra by modulating trait‐dependent species losses and gains. Nature Communications 8: 419. 10.1038/s41467-017-00554-z PMC5583392 28871154

[nph70827-bib-0042] Laughlin DC . 2014. Applying trait‐based models to achieve functional targets for theory‐driven ecological restoration. Ecology Letters 17: 771–784. 24766299 10.1111/ele.12288

[nph70827-bib-0043] Lavorel S , Garnier E . 2002. Predicting changes in community composition and ecosystem functioning from plant traits: revisiting the Holy Grail. Functional Ecology 16: 545–556.

[nph70827-bib-0044] Lefcheck JS . 2016. piecewisesem: piecewise structural equation modelling in R for ecology, evolution, and systematics. Methods in Ecology and Evolution 7: 573–579.

[nph70827-bib-0045] Lepš J , de Bello F , Smilauer P , Dolezal J . 2011. Community trait response to environment: disentangling species turnover vs intraspecific trait variability effects. Ecography 34: 856–863.

[nph70827-bib-0046] Liao J , Quan Q , Ma F , Peng J , Niu S . 2024. Plant height bridges hierarchical community responses to nitrogen enrichment. Journal of Ecology 112: 2069–2081.

[nph70827-bib-0047] Lind EM , Borer E , Seabloom E , Adler P , Bakker JD , Blumenthal DM , Crawley M , Davies K , Firn J , Gruner DS *et al*. 2013. Life‐history constraints in grassland plant species: a growth‐defence trade‐off is the norm. Ecology Letters 16: 513–521. 23347060 10.1111/ele.12078

[nph70827-bib-0048] Maitner BS , Halbritter AH , Telford RJ , Strydom T , Chacon J , Lamanna C , Sloat LL , Kerkhoff AJ , Messier J , Rasmussen N *et al*. 2023. Bootstrapping outperforms community‐weighted approaches for estimating the shapes of phenotypic distributions. Methods in Ecology and Evolution 14: 2592–2610.

[nph70827-bib-0050] McNaughton SJ , Oesterheld M , Frank DA , Williams KJ . 1989. Ecosystem‐level patterns of primary productivity and herbivory in terrestrial habitats. Nature 341: 142–144. 2779651 10.1038/341142a0

[nph70827-bib-0051] Mitchell RM , Bakker JD . 2016. Intraspecific trait variability driven by plasticity and ontogeny in response to grazing. Ecology 97: 3293–3303.27912008

[nph70827-bib-0052] Oksanen J , Blanchet FG , Friendly M , Kindt R , Legendre P , McGlinn D , Minchin PR , O'Hara RB , Simpson GL , Solymos P *et al*. 2024. vegan: community ecology package . R package v.2.6‐8. [WWW document] URL https://CRAN.R‐project.org/package=vegan.

[nph70827-bib-0053] Perez‐Harguindeguy N , Diaz S , Garnier E , Lavorel S , Poorter H , Jaureguiberry P , Bret‐Harte MS , Cornwell WK , Craine JM , Gurvich DE *et al*. 2013. New handbook for standardised measurement of plant functional traits worldwide. Australian Journal of Botany 61: 167–234.

[nph70827-bib-0054] Pérez‐Ramos IM , Matías L , Gómez‐Aparicio L , Godoy Ó . 2019. Functional traits and phenotypic plasticity modulate species coexistence across contrasting climatic conditions. Nature Communications 10: 2555. 10.1038/s41467-019-10453-0 PMC6560116 31186418

[nph70827-bib-0055] Pichon NA , Cappelli SL , Allan E . 2022. Intraspecific trait changes have large impacts on community functional composition but do not affect ecosystem function. Journal of Ecology 110: 644–658.

[nph70827-bib-0056] Pinheiro J , Bates D , DebRoy S , Sarkar D , R Core Team . 2024. nlme: linear and nonlinear mixed effects models . R package v.3.1‐165. [WWW document] URL https://CRAN.R‐project.org/package=nlme.

[nph70827-bib-0057] Pirinen P , Simola H , Aalto J , Kaukoranta JP , Karlsson P , Ruuhela R . 2012. Climatological statistics of Finland 1981–2010 . Finnish Meteorological Institute. Reports 2012:1. 83 pp. Helsinki.

[nph70827-bib-0058] R Core Team . 2024. R: a language and environment for statistical computing. Vienna, Austria: R Foundation for Statistical Computing.

[nph70827-bib-0059] Reich PB . 2014. The world‐wide ‘fast‐slow’ plant economics spectrum: a traits manifesto. Journal of Ecology 102: 275–301.

[nph70827-bib-0060] Salminen JP , Karonen M . 2011. Chemical ecology of tannins and other phenolics: we need a change in approach. Functional Ecology 25: 325–338.

[nph70827-bib-0061] Schiestl FP . 2014. Herbivory and floral signaling: phenotypic plasticity and tradeoffs between reproduction and indirect defense. New Phytologist 204: 313–317. 10.1111/nph.12783 24684288

[nph70827-bib-0062] Shay PE , Trofymow JA , Constabel CP . 2017. An improved butanol‐HCl assay for quantification of water‐soluble, acetone: methanol‐soluble, and insoluble proanthocyanidins (condensed tannins). Plant Methods 13: 63. 28775761 10.1186/s13007-017-0213-3 PMC5539752

[nph70827-bib-0080] Siefert A , Ritchie ME . 2016. Intraspecific trait variation drives functional responses of old‐field plant communities to nutrient enrichment. Oecologia 181: 245–255. doi: 10.1007/s00442-016-3563-z. 26826004

[nph70827-bib-0063] Siefert A , Violle C , Chalmandrier L , Albert CH , Taudiere A , Fajardo A , Aarssen LW , Baraloto C , Carlucci MB , Cianciaruso MV *et al*. 2015. A global meta‐analysis of the relative extent of intraspecific trait variation in plant communities. Ecology Letters 18: 1406–1419. 26415616 10.1111/ele.12508

[nph70827-bib-0064] Speed JDM , Austrheim G , Hester AJ , Mysterud A . 2014. Grazing interacts with climate to determine tree regeneration in alpine ecosystems. Oecologia 175: 97–107.

[nph70827-bib-0065] Stotz GC , Salgado‐Luarte C , Escobedo VM , Valladares F , Gianoli E . 2021. Global trends in phenotypic plasticity of plants. Ecology Letters 24: 2267–2281. 34216183 10.1111/ele.13827

[nph70827-bib-0066] Tatarko AR , Knops JMH . 2018. Nitrogen addition and ecosystem functioning: both species abundances and traits alter community structure and function. Ecosphere 9: e02087.

[nph70827-bib-0067] Telford R , Halbritter A , Maitner B . 2023. traitstrap: bootstrap trait values to calculate moments . R package v.0.1.0. [WWW document] URL https://CRAN.R‐project.org/package=traitstrap.

[nph70827-bib-0068] Tilman D . 1988. Plant strategies and the dynamics and structure of plant communities. Princeton, NJ, USA: Princeton University Press.

[nph70827-bib-0069] Violle C , Enquist BJ , McGill BJ , Jiang L , Albert CH , Hulshof C , Jung V , Messier J . 2012. The return of the variance: intraspecific variability in community ecology. Trends in Ecology & Evolution 27: 244–252. 22244797 10.1016/j.tree.2011.11.014

[nph70827-bib-0070] Wang CN , Li X , Lu XM , Wang Y , Bai YF . 2023. Intraspecific trait variation governs grazing‐induced shifts in plant community above‐and below‐ground functional trait composition. Agriculture, Ecosystems & Environment 346: 108357.

[nph70827-bib-0071] Wang XY , Zhang JJ , Yan XB , Huang KL , Luo X , Zhang YY , Zhou LY , Yang F , Xu XH , Zhou XH *et al*. 2022. Nitrogen enrichment and warming shift community functional composition via distinct mechanisms: The role of intraspecific trait variability and species turnover. Functional Ecology 36: 1230–1242.

[nph70827-bib-0072] War AR , Paulraj MG , Ahmad T , Buhroo AA , Hussain B , Ignacimuthu S , Sharma HC . 2012. Mechanisms of plant defense against insect herbivores. Plant Signaling & Behavior 7: 1306–1320. 22895106 10.4161/psb.21663 PMC3493419

[nph70827-bib-0073] Worm B , Lotze HK , Hillebrand H , Sommer U . 2002. Consumer versus resource control of species diversity and ecosystem functioning. Nature 417: 848–851. 12075351 10.1038/nature00830

[nph70827-bib-0074] Wright IJ , Reich PB , Westoby M , Ackerly DD , Baruch Z , Bongers F , Cavender‐Bares J , Chapin T , Cornelissen JHC , Diemer M *et al*. 2004. The worldwide leaf economics spectrum. Nature 428: 821–827. 15103368 10.1038/nature02403

[nph70827-bib-0075] Xiao Y , Liu X , Zhang L , Song ZP , Zhou SR . 2021. The allometry of plant height explains species loss under nitrogen addition. Ecology Letters 24: 553–562. 33423373 10.1111/ele.13673

[nph70827-bib-0076] Zhang L , Liu X , Zhou S , Shipley B . 2022. Explaining variation in productivity requires intraspecific variability in plant height among communities. Journal of Plant Ecology 15: 310–319.

[nph70827-bib-0077] Zheng SX , Chi YG , Yang XJ , Li WH , Lan ZC , Bai YF . 2022. Direct and indirect effects of nitrogen enrichment and grazing on grassland productivity through intraspecific trait variability. Journal of Applied Ecology 59: 598–610.

[nph70827-bib-0078] Zhou XL , Dong LW , Zhang YJ , Li JD , Ren ZW , Niu KC . 2024. Trait‐dependent importance of intraspecific variation relative to species turnover in determining community functional composition following nutrient enrichment. Oecologia 205: 107–119. 38698244 10.1007/s00442-024-05555-6

[nph70827-bib-0079] Zhou XL , Guo Z , Zhang PF , Du GZ . 2018. Shift in community functional composition following nitrogen fertilization in an alpine meadow through intraspecific trait variation and community composition change. Plant and Soil 431: 289–302.

