# Supplementary material for: Nutrient addition and herbivore exclusion alter plant traits and biomass via distinct mechanisms: intraspecific variability vs species turnover
Source: New Phytol. 2025 Dec 17;249(5):2251–62. doi: 10.1111/nph.70827 (PMC12873523; doi:10.1111/nph.70827)
Supplement: Supplementary file 1 — Fig. S1 Plant community trait composition of plots across all treatments in the low‐productivity and high‐productivity grassland by nonmetric multidimensional scaling analysis, in two‐dimensional space. Fig. S2 Responses of community‐level trait variations induced by interspecific trait variations to nutrient addition and herbivore exclusion by fence in the low‐productivity and the high‐productivity grassland. Fig. S3 Responses of community‐level trait variations induced by intraspecific trait variations to nutrient addition and herbivore exclusion by fence in the low‐productivity and the high‐productivity grassland. Table S1 Results of the linear mixed‐effects models testing effects of nutrient addition and herbivore exclusion by fence on community weight mean of functional traits for the low‐productivity and the high‐productivity grassland separately. Table S2 Results of the permutation test of the effects of nutrient addition and herbivore exclusion by fence on plant community trait composition for the low‐productivity and the high‐productivity grassland separately. Table S3 Results of the linear mixed‐effects models testing effects of nutrient addition and herbivore exclusion by fence on community‐level trait variations induced by interspecific variations for the low‐productivity and the high‐productivity grassland separately. Table S4 Results of the linear mixed‐effects models testing effects of nutrient addition and herbivore exclusion by fence on community‐level trait variations induced by intraspecific variations for the low‐productivity and the high‐productivity grassland separately. Table S5 Results of the linear mixed‐effects models testing effects of nutrient addition and herbivore exclusion by fence on total community biomass for the low‐productivity and the high‐productivity grassland separately. Please note: Wiley is not responsible for the content or functionality of any Supporting Information supplied by the authors. Any queries (other than missing [file NPH-249-2251-s001.docx]

**Article title:** Nutrient addition and herbivore exclusion alter plant traits and biomass via distinct mechanisms: intraspecific variability vs. species turnover

**Authors:** Xuebin Yan; Risto Virtanen; Anu Eskelinen

**Article acceptance date:** 24 November 2025

**Figure S1** Plant community trait composition (i.e., community-weighted trait means) of plots across all treatments in the low-productivity (a) and high-productivity (b) grassland by nonmetric multidimensional scaling (NMDS) analysis, in two-dimensional space.


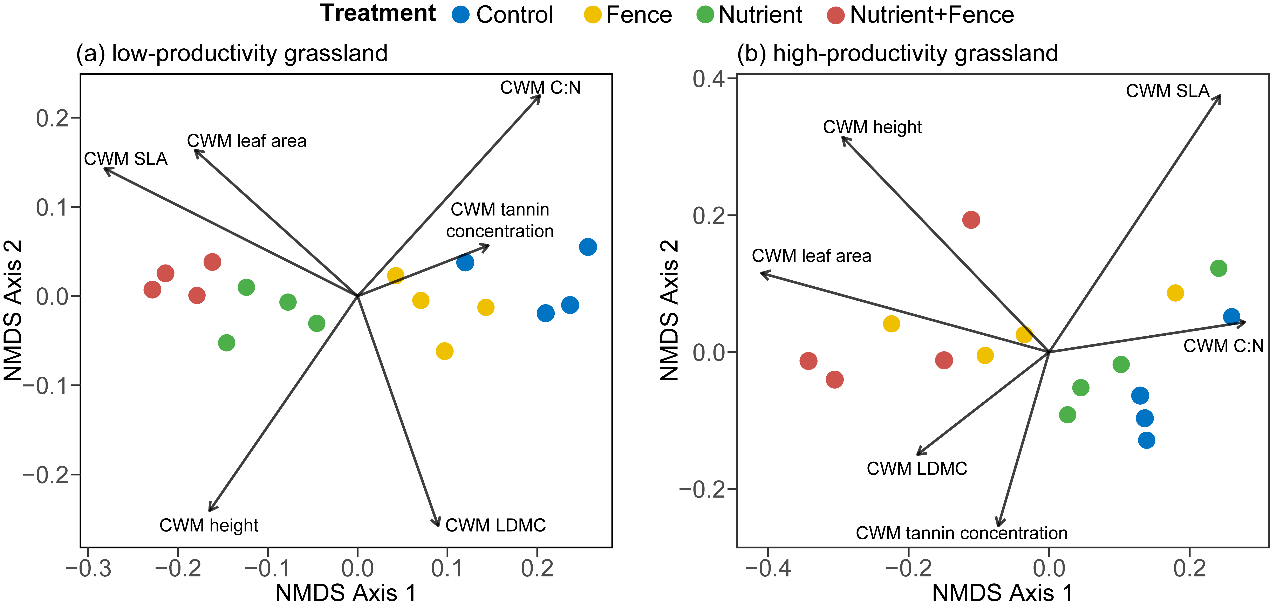


**Figure S2** Responses of community level trait variations induced by interspecific trait variations (CWM_fixed_) to nutrient addition and herbivore exclusion by fence in the low-productivity and the high-productivity grassland. Dots with deep colors represent the mean CWM values of each treatment, and points with light colors represent the raw CWM values of each plot. Error bars represent +/- SE.


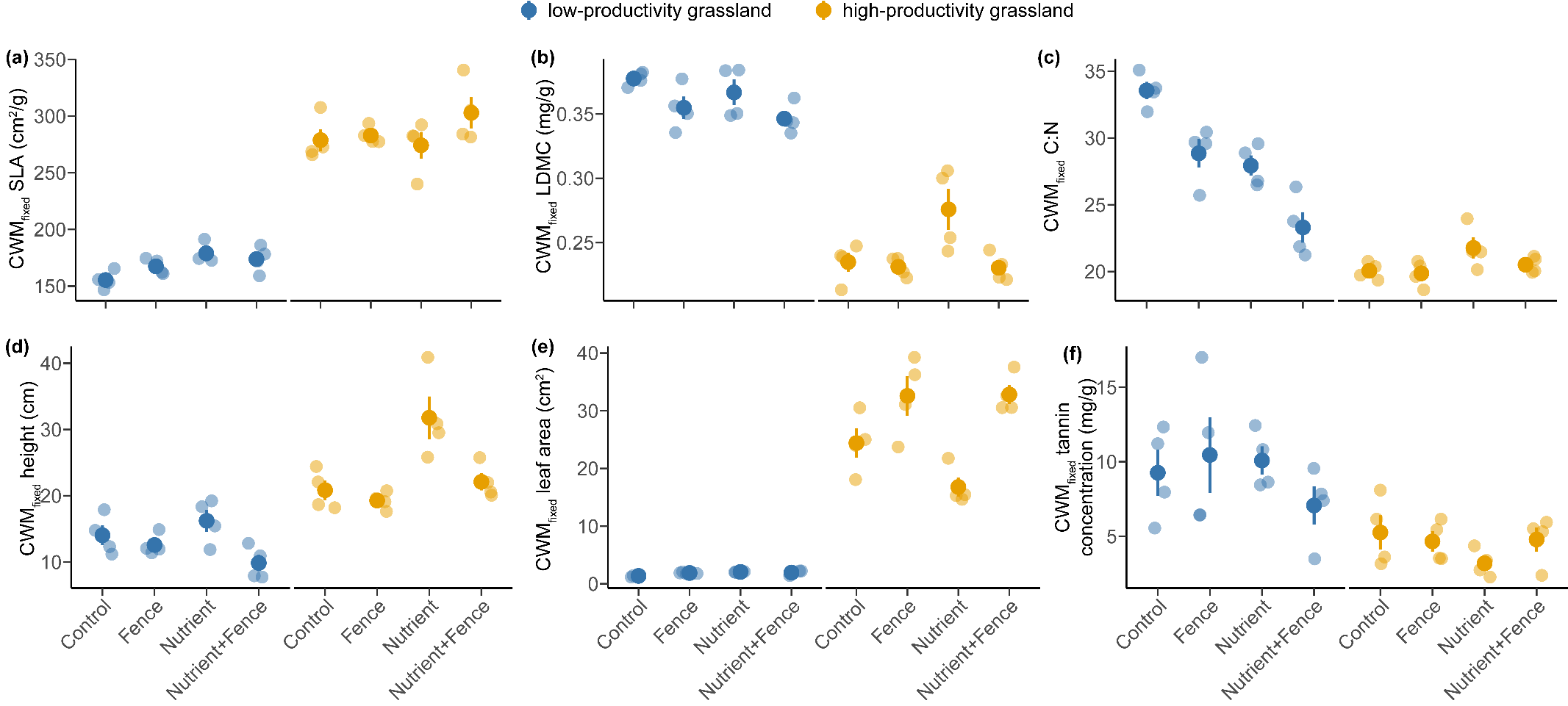


**Figure S3** Responses of community level trait variations induced by intraspecific trait variations (ITV) to nutrient addition and herbivore exclusion by fence in the low-productivity and the high-productivity grassland. Dots with deep colors represent the mean CWM values of each treatment, and points with light colors represent the raw CWM values of each plot. Error bars represent +/- SE.


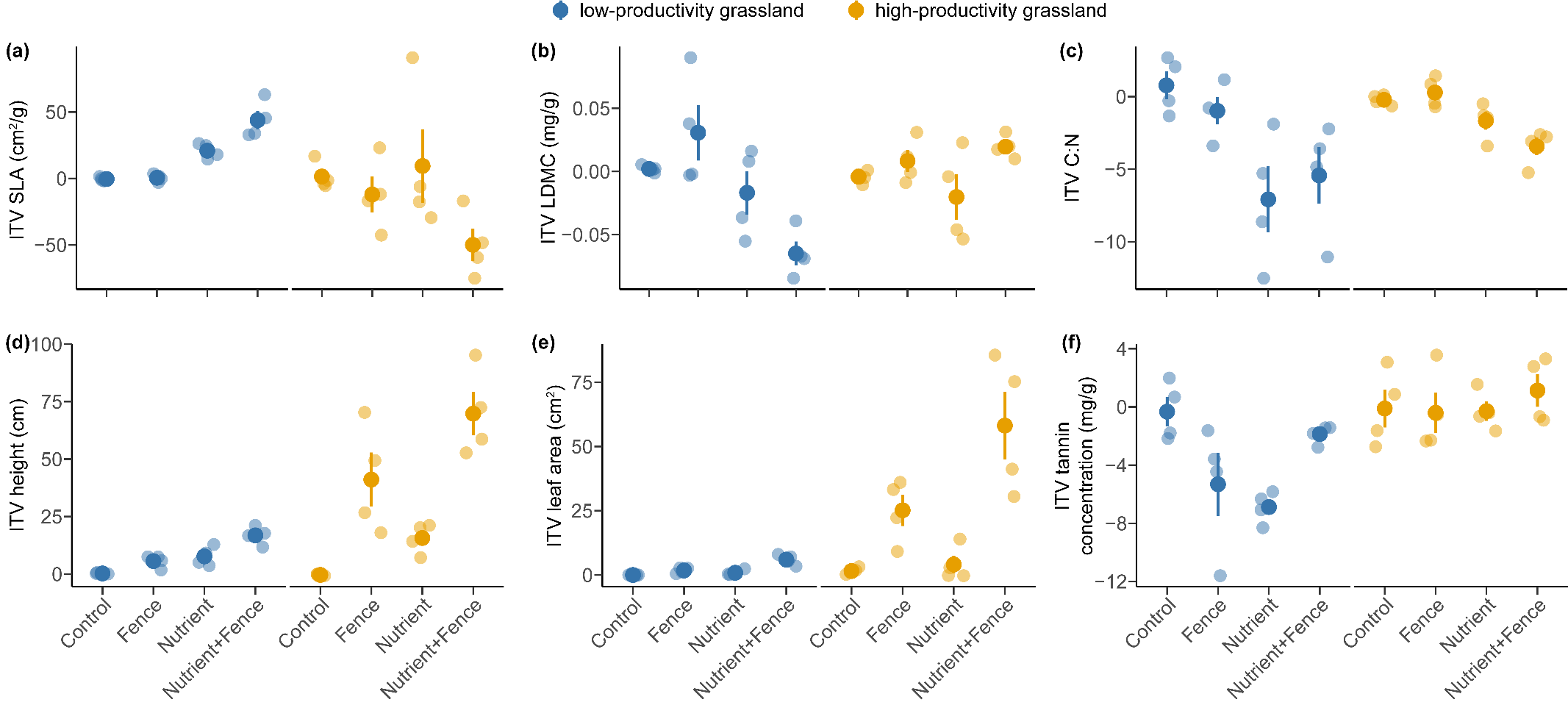


**Table S1** Results of the linear mixed effect models testing effects of nutrient addition and herbivore exclusion by fence on community weight mean (CWM) of functional traits for the low-productivity and the high-productivity grassland separately. num*DF* and den*DF* denote the numerator and denominator degrees of freedom, respectively. Significant intercepts and fixed effects (*P* < 0.05) are in bold and marginally significant intercepts and fixed effects (0.05 ≤ *P* < 0.1) are in italics. CWM height, leaf area and tannin concentration were log transformed to meet the normality.

|  |  |  |  | **CWM SLA** | |  | **CWM LDMC** | |  | **CWM C: N** | |  | **CWM height** | |  | **CWM leaf area** | |  | **CWM tannin concentration** | |
| --- | --- | --- | --- | --- | --- | --- | --- | --- | --- | --- | --- | --- | --- | --- | --- | --- | --- | --- | --- | --- |
| **Habitats** | **Fixed effects** | **Num*DF*** | **Den*DF*** | ***F*** | ***P*** |  | ***F*** | ***P*** |  | ***F*** | ***P*** |  | ***F*** | ***P*** |  | ***F*** | ***P*** |  | ***F*** | ***P*** |
| low-productivity grassland | Intercept | 1 | 9 | 5686.85 | <0.01 |  | 1574.00 | <0.01 |  | 1447.65 | <0.01 |  | 185.92 | <0.01 |  | 84.18 | <0.01 |  | 26.67 | <0.01 |
|  | N | 1 | 9 | 243.63 | **<0.01** |  | 18.97 | **<0.01** |  | 78.29 | **<0.01** |  | 34.25 | **<0.01** |  | 21.86 | **<0.01** |  | 4.61 | *0.06* |
|  | F | 1 | 9 | 25.81 | **<0.01** |  | 4.13 | *0.07* |  | 12.66 | **0.01** |  | 4.68 | *0.06* |  | 34.03 | **<0.01** |  | 0.46 | 0.51 |
|  | N×F | 1 | 9 | 0.63 | 0.45 |  | 5.87 | **0.04** |  | 1.73 | 0.22 |  | 0.14 | 0.72 |  | 4.78 | 0.06 |  | 4.81 | *0.06* |
| high productivity grassland | Intercept | 1 | 9 | 1163.48 | <0.01 |  | 3171.96 | <0.01 |  | 1440.93 | <0.01 |  | 189.69 | <0.01 |  | 138.61 | <0.01 |  | 19.10 | <0.01 |
|  | N | 1 | 9 | 0.21 | 0.66 |  | 4.22 | *0.07* |  | 3.10 | 0.11 |  | 13.38 | **0.01** |  | 3.26 | 0.10 |  | 0.04 | 0.85 |
|  | F | 1 | 9 | 1.59 | 0.24 |  | 0.03 | 0.86 |  | 2.92 | 0.12 |  | 27.83 | **<0.01** |  | 42.78 | **<0.01** |  | 0.51 | 0.49 |
|  | N×F | 1 | 9 | 0.45 | 0.52 |  | 0.68 | 0.43 |  | 4.41 | *0.07* |  | 0.08 | 0.78 |  | 6.03 | **0.04** |  | 1.71 | 0.22 |

**Table S2** Results of the permutation test of the effects of nutrient addition and herbivore exclusion by fence on plant community trait composition (community weighted trait means) for the low-productivity and the high-productivity grassland separately. *DF* and SS denote degrees of freedom and sums of squares, respectively. Significant results (*P* < 0.05) are in bold and marginally significant results (0.05 ≤ *P* < 0.1) are in italics.

| **Habitats** | **Fixed effects** | ***DF*** | **SS** | ***R^2^*** | ***F*** | ***P*** |
| --- | --- | --- | --- | --- | --- | --- |
| low-productivity grassland | N | 1 | 0.09 | 0.76 | 80.92 | **<0.01** |
|  | F | 1 | 0.01 | 0.11 | 11.68 | **<0.01** |
|  | N×F | 1 | <0.01 | 0.01 | 1.50 | 0.22 |
|  | Residual | 12 | 0.01 | 0.11 |  |  |
|  | Total | 15 | 0.12 | 1.00 |  |  |
| high-productivity grassland | N | 1 | 0.02 | 0.11 | 3.62 | *0.06* |
|  | F | 1 | 0.06 | 0.48 | 15.48 | **<0.01** |
|  | N×F | 1 | <0.01 | 0.04 | 1.23 | 0.28 |
|  | Residual | 12 | 0.07 | 0.37 |  |  |
|  | Total | 15 | 0.18 | 1.00 |  |  |

**Table S3** Results of the linear mixed effect models testing effects of nutrient addition and herbivore exclusion by fence on community-level trait variations induced by interspecific variations (CWM_fixed_) for the low-productivity and the high-productivity grassland separately. num*DF* and den*DF* denote the numerator and denominator degrees of freedom, respectively. Significant intercepts and fixed effects (*P* < 0.05) are in bold and marginally significant intercepts and fixed effects (0.05 ≤ *P* < 0.1) are in italics. CWM_fixed_ of height, leaf area and tannin concentration were log transformed to meet the normality.

|  |  |  |  | **CWM_fixed_ SLA** | |  | **CWM_fixed_ LDMC** | |  | **CWM_fixed_ C: N** | |  | **CWM_fixed_ height** | |  | **CWM_fixed_ leaf area** | |  | **CWM_fixed_ tannin concentration** | |
| --- | --- | --- | --- | --- | --- | --- | --- | --- | --- | --- | --- | --- | --- | --- | --- | --- | --- | --- | --- | --- |
| **Habitats** | **Fixed effects** | **Num*DF*** | **Den*DF*** | ***F*** | ***P*** |  | ***F*** | ***P*** |  | ***F*** | ***P*** |  | ***F*** | ***P*** |  | ***F*** | ***P*** |  | ***F*** | ***P*** |
| low-productivity grassland | Intercept | 1 | 9 | 2646.13 | <0.01 |  | 4434.86 | <0.01 |  | 1803.77 | <0.01 |  | 376.33 | <0.01 |  | 600.18 | <0.01 |  | 47.42 | <0.01 |
|  | N | 1 | 9 | 19.08 | **<0.01** |  | 2.90 | 0.12 |  | 56.80 | **<0.01** |  | 0.04 | 0.84 |  | 17.14 | **<0.01** |  | 1.17 | 0.31 |
|  | F | 1 | 9 | 1.02 | 0.34 |  | 14.46 | **<0.01** |  | 39.64 | **<0.01** |  | 8.78 | **0.02** |  | 4.55 | *0.06* |  | 0.59 | 0.46 |
|  | N×F | 1 | 9 | 6.27 | **0.03** |  | 0.04 | 0.85 |  | <0.01 | 0.96 |  | 3.43 | 0.10 |  | 11.79 | **0.01** |  | 3.13 | 0.11 |
| high-productivity grassland | Intercept | 1 | 9 | 3011.10 | <0.01 |  | 2702.83 | <0.01 |  | 6445.57 | <0.01 |  | 603.60 | <0.01 |  | 482.14 | <0.01 |  | 120.42 | <0.01 |
|  | N | 1 | 9 | 0.55 | 0.48 |  | 4.64 | *0.06* |  | 5.31 | **0.05** |  | 12.92 | **0.01** |  | 2.31 | 0.16 |  | 1.42 | 0.26 |
|  | F | 1 | 9 | 2.49 | 0.15 |  | 6.90 | **0.03** |  | 2.00 | 0.19 |  | 8.61 | **0.02** |  | 24.85 | **<0.01** |  | 0.38 | 0.55 |
|  | N×F | 1 | 9 | 1.40 | 0.27 |  | 4.98 | **0.05** |  | 1.08 | 0.33 |  | 4.50 | *0.06* |  | 2.62 | 0.14 |  | 1.82 | 0.21 |

**Table S4** Results of the linear mixed effect models testing effects of nutrient addition (N) and herbivore exclusion by fence (F) on community-level trait variations induced by intraspecific variations (ITV) for the low-productivity and the high-productivity grassland separately. num*DF* and den*DF* denote the numerator and denominator degrees of freedom, respectively. Significant intercepts and fixed effects (*P* < 0.05) are in bold and marginally significant intercepts and fixed effects (0.05 ≤ *P* < 0.1) are in italics.

|  |  |  |  | **ITV SLA** | |  | **ITV LDMC** | |  | **ITV C: N** | |  | **ITV height** | |  | **ITV leaf area** | |  | **ITV tannin concentration** | |
| --- | --- | --- | --- | --- | --- | --- | --- | --- | --- | --- | --- | --- | --- | --- | --- | --- | --- | --- | --- | --- |
| **Habitats** | **Fixed effects** | **Num*DF*** | **Den*DF*** | ***F*** | ***P*** |  | ***F*** | ***P*** |  | ***F*** | ***P*** |  | ***F*** | ***P*** |  | ***F*** | ***P*** |  | ***F*** | ***P*** |
| low-productivity grassland | Intercept | 1 | 9 | 71.26 | <0.01 |  | 2.78 | 0.13 |  | 14.38 | <0.01 |  | 52.77 | <0.01 |  | 29.09 | <0.01 |  | 29.51 | <0.01 |
|  | N | 1 | 9 | 70.57 | **<0.01** |  | 15.04 | **<0.01** |  | 14.31 | **<0.01** |  | 46.35 | **<0.01** |  | 19.95 | **<0.01** |  | 1.66 | 0.23 |
|  | F | 1 | 9 | 9.62 | **0.01** |  | 0.43 | 0.53 |  | 0.00 | 0.97 |  | 28.21 | **<0.01** |  | 36.68 | **<0.01** |  | 0.00 | 0.99 |
|  | N×F | 1 | 9 | 8.18 | **0.02** |  | 6.75 | **0.03** |  | 1.10 | 0.32 |  | 1.89 | 0.20 |  | 8.66 | **0.02** |  | 17.22 | **<0.01** |
| high-productivity grassland | Intercept | 1 | 9 | 2.32 | 0.16 |  | 0.02 | 0.88 |  | 23.51 | <0.01 |  | 66.79 | <0.01 |  | 25.16 | <0.01 |  | 0.01 | 0.93 |
|  | N | 1 | 9 | 0.80 | 0.39 |  | 0.05 | 0.82 |  | 25.49 | **<0.01** |  | 8.40 | **0.02** |  | 6.52 | **0.03** |  | 0.59 | 0.46 |
|  | F | 1 | 9 | 4.74 | *0.06* |  | 6.58 | **0.03** |  | 1.52 | 0.25 |  | 38.32 | **<0.01** |  | 31.20 | **<0.01** |  | 0.41 | 0.54 |
|  | N×F | 1 | 9 | 1.88 | 0.20 |  | 1.81 | 0.21 |  | 4.94 | *0.05* |  | 0.67 | 0.43 |  | 4.78 | *0.06* |  | 0.95 | 0.36 |

**Table S5** Results of the linear mixed effect models testing effects of nutrient addition and herbivore exclusion by fence on total community biomass for the low-productive and the high-productivity grassland separately. num*DF* and den*DF* denote the numerator and denominator degrees of freedom, respectively. Significant results (*P* < 0.05) are in bold and marginally significant results (0.05 ≤ *P* < 0.1) are in italics. The biomass data was log-transformed to meet the normality.

| **Habitats** | **Fixed effects** | **num*DF*** | **den*DF*** | ***F*** | ***P*** |
| --- | --- | --- | --- | --- | --- |
| low-productivity grassland | Intercept | 1 | 9 | 1201.55 | **<0.01** |
|  | N | 1 | 9 | 37.77 | **<0.01** |
|  | F | 1 | 9 | 4.72 | *0.06* |
|  | N×F | 1 | 9 | 0.96 | 0.35 |
| High-productivity grassland | Intercept | 1 | 9 | 2705.32 | **<0.01** |
|  | N | 1 | 9 | 1.48 | 0.25 |
|  | F | 1 | 9 | 7.07 | **0.03** |
|  | N×F | 1 | 9 | 0.00 | 0.98 |
